# Supplementary material for: Effect of Solution Treatment Time on Microstructure Evolution and Properties of Mg-3Y-4Nd-2Al Alloy
Source: Materials (Basel). 2023 Mar 22;16(6):2512. doi: 10.3390/ma16062512 (PMC10052546; doi:10.3390/ma16062512)
Supplement: Supplementary file 1 [file materials-16-02512-s001.zip › materials-2271180-supplementary.pdf]

Supporting Information

Effect of Solution Treatment Time on Microstructure Evolution and Properties of Mg-3Y-4Nd-2Al Alloy

Lili Zhao[1,2,3], Sicong Zhao[1,2,*], Yicheng Feng[1,2], Lei Wang[1,2], Rui Fan[1,2],Tao Ma [1,2] and Liping Wang[1,2,*]

[1]*School of Material Science and Chemical Engineering, Harbin University of Science and Technology, Harbin 150000, China*

[2]*Key Laboratory of Advanced Manufacturing and Intelligent Technology (Ministry of Education), Harbin University of Science and Technology, Harbin 150000, China*

[3]*College of Light Industry, Harbin University of Commerce, Harbin, 150028, Heilongjiang, China*

**Correspondence: zscwr@163.com (S.Z.); lp_wang2003@126.com (L.W.)*

The EDS results

As shown in Figure S1, the total amount of RE elements in the matrix of as-cast alloy is 0.25 at%, and the total amount of RE elements in the matrix after the solution treatment is 0.62 at%. Thus, the solid solute elements in the matrix increased after the solution treatment.

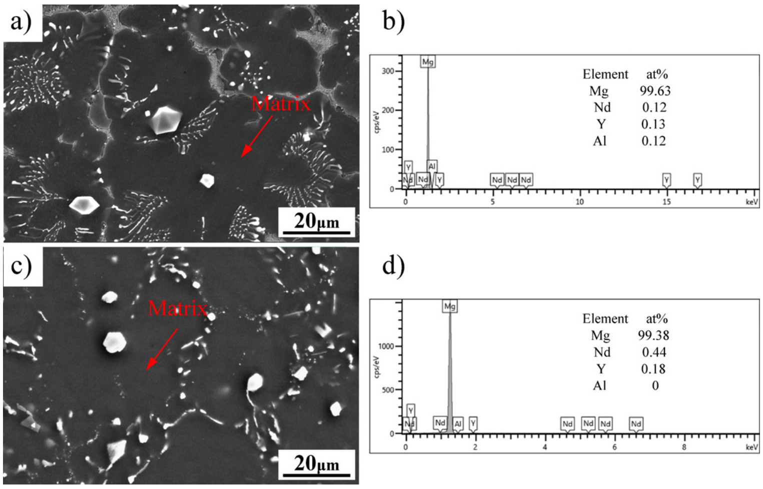

Figure S1. SEM and EDS results of α-Mg matrix, a) and b)as-cast alloy; c) and d)solution-treated alloy.
